# Impact of Oxygen Vacancies in LiCoO_2_ on the Electrochemical Performance of Garnet‐Based All‐Solid‐State Li‐Metal Batteries

**DOI:** 10.1002/advs.202508750

**Published:** 2025-07-25

**Authors:** Zhizhen Qin, Jehad Ahmed, Sebastian Speer, Dmitri L. Danilov, Anna Windmüller, Shicheng Yu, Chih‐Long Tsai, Hermann Tempel, Josef Granwehr, Wen‐Wei Wu, Jeng‐Kuei Chang, Rüdiger‐A. Eichel, Peter H. L. Notten

**Affiliations:** ^1^ Institute of Energy Technologies: Fundamental Electrochemistry (IET‐1) Forschungszentrum Jülich GmbH 52428 Jülich Germany; ^2^ Institute of Physical Chemistry RWTH Aachen University 52074 Aachen Germany; ^3^ Eindhoven University of Technology P.O. Box 513 Eindhoven MB 5600 The Netherlands; ^4^ Department of Materials Science and Engineering National Yang Ming Chiao Tung University Hsinchu 30010 Taiwan; ^5^ Institute of Energy Materials and Devices Helmholtz Institute Münster: Ionics in Energy Storage (IMD‐4/HI MS) Forschungszentrum Jülich 48149 Münster Germany; ^6^ University of Technology Sydney Broadway Sydney NSW 2007 Australia

**Keywords:** all‐solid‐state Li batteries, composite cathode, garnet, LiCoO_2_, LLZO

## Abstract

Garnet‐structured Li_7_La_3_Zr_2_O_7_ (LLZO) is considered as one of the most promising solid electrolytes for high safety all‐solid‐state Li batteries (SSLBs) applications. However, this type of SSLB utilizing LiCoO_2_/LLZO as composite cathode faces high capacity degradation because of delamination between LiCoO_2_ (LCO) and LLZO and possible oxygen vacancy‐driven microcrack formation within LCO. Herein, a pure oxygen atmosphere is used for sintering the composite cathode to limit oxygen vacancy formation in LCO. Different sintering temperatures are also used to reduce the effect of sintering atmospheres, which suggests the non‐reversible oxidation peak at ∼3.8 V is not related to Li_2_CO_3_ formation. Although the Coulombic efficiencies of the first electrochemical cycle of SSLBs sintered in pure oxygen atmosphere are improved, their electrochemical performances are lower than that of air‐sintered SSLB due to higher cell resistances from the reduction of oxygen vacancies in LCO and possible higher volume change during electrochemical cycling. Also, the lower electrochemical cycling performance and observing tens of micrometers long inter‐granular cracks in the highly dense composite cathode suggests that microstructural optimization is more important than a high relative density. These observations provide guidelines for further improving the electrochemical cycling performance of garnet‐structure‐based SSLBs toward practical applications.

## Graphical abstract

Slight oxygen vacancy formation in LiCoO_2_ increases its electron charge carrier and Co^2+^/Co^3+^ mixed valence states to enhance its total conductivity, thereby increasing the performance of an All‐solid‐state Li battery based on LiCoO_2_ as the cathode and garnet‐structured LLZO as the solid electrolyte.

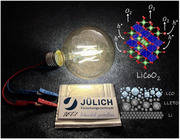

## 1 Introduction

Ever since commercialization in 1991, lithium‐ion batteries (LIBs) have gradually developed into the most widely used energy storage devices in portable electronics and electric vehicles (EVs).^[^ ^1^ ^]^ However, the state‐of‐the‐art LIBs have specific energy densities of ≈265 to 280 Wh kg^−1^, which are already approaching their theoretical limits.^[^ ^2^, ^3^ ^]^ The demand for energy densities higher than 500 Wh kg^−1^ urges the development of next‐generation high energy density batteries (HEDB) for EV and humanoid robotic applications.^[^ ^4^ ^]^

Li metal is a promising anode material for HEDB because of its low electrochemical potential (−3.04 V vs. Standard Hydrogen Electrode), ultrahigh specific capacity (3860 mAh g^−1^), and low density (0.53 g cm^−3^).^[^ ^5^ ^]^ However, the combination of typical liquid electrolytes and Li metal anodes causes many problems. First, Li metal reacts with the liquid electrolyte to form a solid electrolyte interface (SEI). This reaction causes a large consumption of both active Li and liquid electrolyte, resulting in a low Coulombic efficiency (CE).^[^ ^6^ ^]^ Second, the huge volume expansion of the Li anode during cycling causes the brittle SEI to rupture, thereby exposing fresh Li surface to the liquid electrolyte to promote the formation of new SEI, which further consumes active Li and liquid electrolyte. Thirdly, uneven Li deposition due to Li‐ion concentration fluctuations leads to the formation of Li dendrites, which also facilitates the side reaction between Li and the liquid electrolytes or Li fall‐off.^[^ ^7^, ^8^ ^]^ The continuous growth of Li dendrites may eventually puncture the separator and cause short circuits.^[^ ^9^ ^]^ Last but not least, the risk of leakage and ease of catching fire pose a safety concern of liquid electrolytes. These challenges have led to active research into possible solutions, such as liquid electrolyte engineering,^[^ ^10^, ^11^, ^12^ ^]^ artificial SEI,^[^ ^13^, ^14^ ^]^ solid electrolytes (SEs) for solid‐state batteries,^[^ ^15^, ^16^ ^]^ and structural design of battery components.^[^ ^17^, ^18^, ^19^ ^]^ Among the possible solutions, solid electrolytes have become an attractive research topic in recent years due to their intrinsic safety and potential for achieving HEDBs.

Due to its wide electrochemical window, good chemical and electrochemical stability to Li metal, and good ionic conductivity, garnet‐structured Li_7_La_3_Zr_2_O_7_‐based (LLZO) SE shows great potential for all‐solid‐state Li battery (SSLB) applications.^[^ ^20^ ^]^ Various efforts have been made to improve its practicality, such as further increasing the ionic conductivity,^[^ ^21^, ^22^ ^]^ reducing the Li/SE interfacial resistance,^[^ ^23^, ^24^ ^]^ and suppressing Li dendrite penetration.^[^ ^25^ ^]^ However, due to the electrochemical and physical nature of the active cathode material (ACM), the breathing behavior of the ACM causes instability of the cathode/SE interface during electrochemical cycling, which becomes the main obstacle for achieving long‐term stability of all‐solid‐state HEDBs. The composite cathode consisting of mixed ACM and SE particles is usually used to establish 3D percolation networks for ionic and electronic transport^[^ ^26^ ^]^ and to overcome the unfavorable effects of the low Li‐ion conductivity of ACMs. Since ACM and LLZO are very rigid, the construction of such 3D composite cathodes often requires sintering at very high temperatures to establish good contacts between LiCoO_2_ (LCO) and LLZO particles within the composite cathode as well as between the composite cathode and SE.^[^ ^27^, ^28^ ^]^ The selection of the ACM material is another important aspect, since the ACM must have high chemical stability against SE at elevated sintering temperatures, high electronic conductivity to provide electronic conduction paths, and a thermal expansion coefficient close to that of LLZO to avoid delamination during sintering. This makes LCO the best ACM material choice for LLZO‐based composite cathode.^[^ ^29^, ^30^ ^]^ Although the cycling stability of LLZO‐based SSLBs has been improved with the above‐mentioned strategies, it is still far from meeting the requirements for practical use. This is mainly due to the fact that the degradation mechanism of the LLZO‐based composite cathode has not yet been fully understood.

Microstructural defects have a significant impact on the capacity degradation of composite cathodes. Based on percolation theory, Bielefeld et al. modeled the impact of structure parameters on the ionic/electronic conductivity of composite cathodes.^[^ ^31^ ^]^ They concluded that higher porosity caused more tortuous paths for ionic and electronic transport, which hindered the electrochemical performance. Further microstructural disintegration during electrochemical cycling of SSLB is also an important reason for rapid capacity fading. Liu et al. observed apparent cracks in the LiCoO_2_ (60 wt.%), Li_3_BO_3_ (30 wt.%), and In_2(1‐x)_Sn_2x_O_3_ (10 wt.%) composite cathode after the SSLB was subjected to high current density cycling. They concluded that these cracks are caused by the volume change of LCO and are responsible for the degradation of this type of SSLB.^[^ ^32^ ^]^ Apart from these mechanical degradations, thermodynamic stability is another important aspect. Miara et al. calculated the reaction energy of LLZO against different cathodes by using density functional theory (DFT). Their results show that the driving force for LiFePO_4_/LLZO, decomposing to LaFeO_3_, La_2_Zr_2_O_7_, Fe_2_O_3_, and Li_3_PO_4_, is relatively high, while that for LCO/LLZO, decomposing to La_2_O_3_, La_2_Zr_2_O_7,_ and Li_2_CO_3_, is low at their typical operating voltages, but still is the reason for SSLB capacity degradation.^[^ ^33^ ^]^

To further understand the capacity degradation mechanism of garnet‐based SSLBs, *in‐operando* transmission electron microscopy (TEM) was used to investigate the electrochemical cycling process of the LCO/LLZO composite cathode.^[^ ^30^ ^]^ Two possible reasons for garnet‐based SSLB degradation were concluded. First, delamination occurred at the LCO/LLZO interface because of the stresses generated by the breathing behavior of LCO during electrochemical cycling. Second, cobalt segregation occurred within LCO grains during electrochemical cycling, leading to the development of microcracks within LCO grains and loss of LCO capacity. This latter observation of cobalt segregation within LCO grains was hypothesized to be due to the presence of oxygen vacancies within LCO lattices during high‐temperature sintering, as suggested by Yaqoob et al. from their first‐principles DFT calculations and microstructural mechanical analysis of LCO/LLZO composite cathode.^[^ ^34^ ^]^ The formation of oxygen vacancies within LCO induces large local strain during electrochemical cycling, which initiates microcracking within LCO. It also facilitated the migration of Co to escape from LCO lattices to form metallic Co and release oxygen during electrochemical cycling.^[^ ^34^, ^35^ ^]^ Thus, it has been proposed that battery capacity degradation may be suppressed by sintering the SSLB under a pure oxygen atmosphere to reduce the formation of oxygen vacancies in LCO.

In this work, the LCO/LLZO composite cathode was sintered onto LLZO SE under a pure oxygen atmosphere to examine the proposed hypothesis. To limit the effect of different sintering atmosphere, i.e. pure oxygen vs. air, different sintering temperatures were also applied to optimize the oxygen atmosphere sintered SSLBs. Subsequently, Raman mapping was used to confirm and identify the individual LCO and LLZO phases to ensure that no obvious chemical reaction took place during the sintering process. Then, the electrochemical performances of the fabricated LLZO‐based SSLBs were characterized to compare their cycling stability. Scanning electron microscopy (SEM) was used to identify the changes in their microstructure by comparing the microstructures before and after cycling to find possible reasons for the lower electrochemical performance of the pure oxygen atmosphere sintered SSLBs compared to air‐sintered ones.

## 2 Results and Discussion

**Figure 1:**
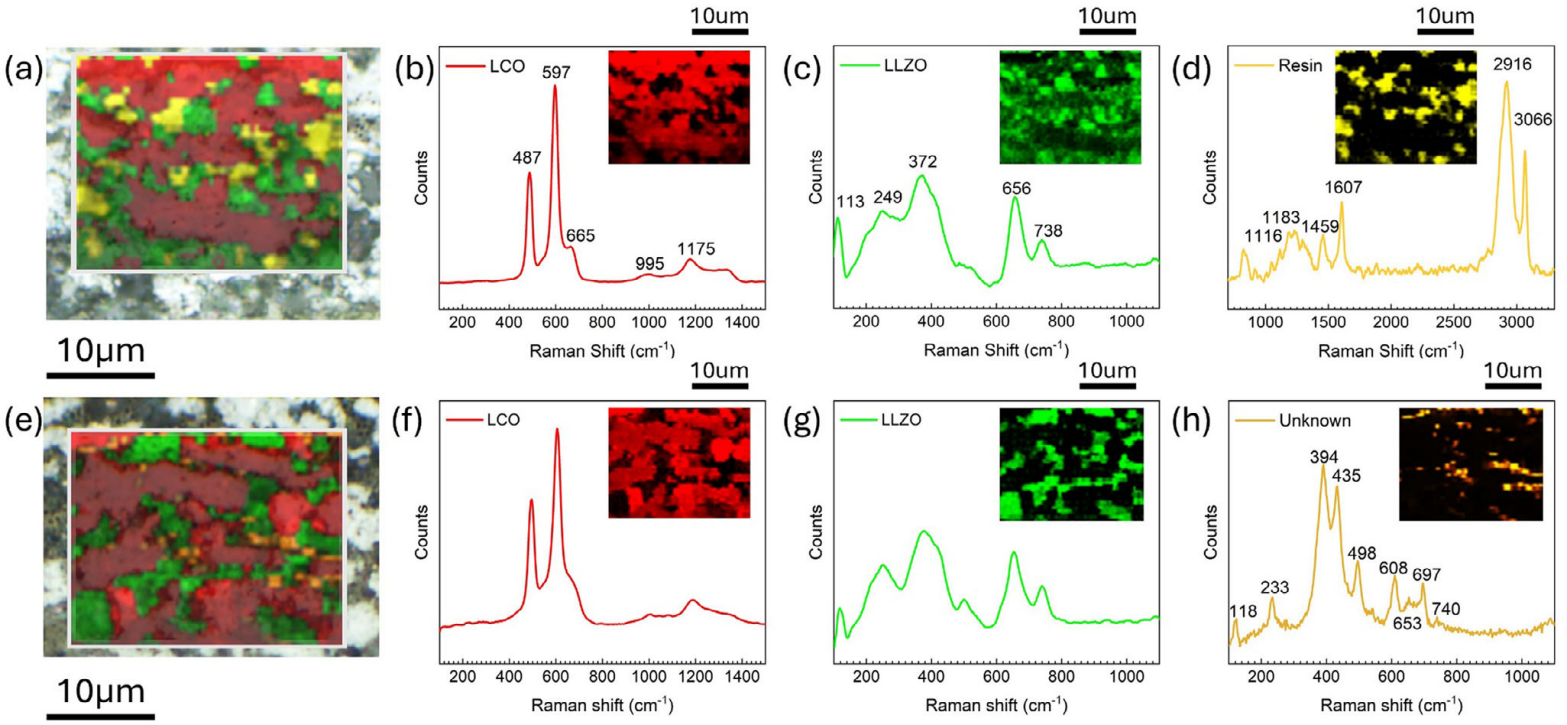
Raman mapping of the sintered composite cathodes for O970 and O1000. The results for O970, showing its a) optical image overlayed with a Raman mapped area, and b–d) analyzed average Raman spectra for LCO, LLZTO, and resin, respectively. The results for O1000, showing its e) optical image overlayed with a Raman mapped area, and f–h)analyzed average spectra for LCO, LLZO, and possible impurity phase, respectively.

To ensure minimum side reactions that may affect the electrochemical performance of the fabricated SSLBs, high resolution Raman mapping was used to identify possible impurities as well as the distribution of LCO and Li_6.45_Al_0.05_La_3_Zr_1.6_Ta_0.4_O_12_ (LLZTO) phases of the composite cathodes sintered at different temperatures. Here, the SSLBs sintered in pure oxygen atmosphere from 950 to 1000 °C with temperature steps of 10 °C are indicated as O950, O960, O970, O980, O990, and O1000, while the reference SSLB A970 was sintered in air at 970 °C. For the high solution Raman mapping, O970 and O1000 are shown as examples in **Figure** **1**. Three phases were identified in the composite cathode of O970, Figure 1b–d, which are LCO, LLZTO, and epoxy resin, respectively. For the LCO phase, Figure 1b, the average spectrum shows three characteristic LCO peaks at 487, 597, and 1175 cm^−1^, which can be attributed to the O‐Co‐O bending mode (E_g_), the Co‐O stretching mode (A_1g_),^[^ ^36^ ^]^ and the overtone of the A_1g_ mode,^[^ ^37^ ^]^ respectively. A peak at 995 cm^−1^ is also shown in a spectrum of LCO in literature,^[^ ^37^ ^]^ but its origin remains unclear. The peak at 665 cm^−1^ can be attributed to the A_1g_ mode of Co_3_O_4_, which should be an impurity contained in pristine LCO.^[^ ^29^ ^]^ The corresponding LLZTO phase is shown in Figure 1c. The appearance of two shoulder peaks at 213 and 415 cm^−1^ and two broad peaks at 249 and 372 cm^−1^ instead of sharp peaks ≈209, 291, 346, 370, and 404 cm^−1^ illustrates that the LLZTO is in the cubic phase instead of tetragonal.^[^ ^38^ ^]^ The peak at 656 cm^−1^ is associated with the vibrational stretching modes of the Zr─O bond for ZrO_6_ octahedra^[^ ^39^ ^]^ while that at 738 cm^−1^ corresponds to the stretching of the Ta‐O bond of the TaO_6_ octahedra, demonstrating the successful substitution of Zr by Ta.^[^ ^40^ ^]^ Epoxy resin was also recognized from its characterization Raman peaks at 2916 and 3066 cm^−1^, Figure 1d, which would represent the porosity in the composite cathode after the sintering process at 970 °C. Similar results were obtained for the sample sintered in air, i.e. A970, from Raman mappings as shown in Figure S1 (Supporting information).

By further increasing the sintering temperature to 1000 °C, O1000 differs from others in its microstructure and chemistry within the composite cathode. For O1000, Figure 1e–h, no resin was detected, indicating the denser microstructure compared to the samples sintered at lower temperatures. In addition to LCO and cubic LLZTO phases, Figure 1f,g, an unknown phase also appears in the mapping, Figure 1h. From the mixed map Figure 1e, it can be seen that this phase appears mainly in the LLZTO region and near the interfaces between LCO and LLZTO. Two major Raman peaks at 394 and 435 cm^−1^ have been identified for this material. By searching these Raman peaks through references, we exclude the possibility of commonly seen impurities in the LLZTO system, including the tetragonal phase LLZO,^[^ ^38^ ^]^ Li containing materials such as Li_2_CO_3_ (1090 cm^−1^),^[^ ^41^ ^]^ Li_2_O (525 cm^−1^),^[^ ^42^, ^43^ ^]^ LiOH (324 cm^−1^),^[^ ^43^ ^]^ LiAlO_2_ (508 cm^−1^);^[^ ^44^ ^]^ La containing materials such as La_2_O_3_ (337 and 442 cm^−1^),^[^ ^45^ ^]^ LaAlO_3_ (490 cm^−1^),^[^ ^46^ ^]^ LaCoO_3_ (657 cm^−1^); Zr containing materials such as ZrO_2_ (cubic‐ZrO_2_ 145 and 246 cm^−1^; tetragonal ZrO_2_‐292 cm^−1^; monoclinic ZrO_2_‐335, 381 and 476 cm^−1^),^[^ ^47^ ^]^ Li_2_ZrO_3_ (383, 476, and 578 cm^−1^)^[^ ^48^ ^]^ and Ta containing material Ta_2_O_5_ (642 cm^−1^).^[^ ^49^ ^]^ Nevertheless, it is possible to be Li_0.5_La_2_Co_0.5_O_4_ (371, 425, and 685 cm^−1^)^[^ ^50^ ^]^ or La_2_Zr_2_O_7_ (300 and 394 cm^−1^),^[^ ^51^ ^]^ with Li_0.5_La_2_Co_0.5_O_4_ having the higher probability because the main Raman peak for La_2_Zr_2_O_7_ at 300 cm^−1^ was not observed in all spectra. Although the relative intensities of the characteristic peaks of Li_0.5_La_2_Co_0.5_O_4_ are not the same as those of the unknown phase found here, their Raman shifts are similar. Therefore, a chemical reaction between LCO and LLZTO at sintering temperature higher than 1000 °C in a pure oxygen atmosphere, possibly to form Li_0.5_La_2_Co_0.5_O_4_, was observed. The selected microstructure and EDS mappings for O950, O970, O1000, and A970 are shown in Figures S2–S5 (Supporting information) to confirm that there were neither severe chemical reactions nor elemental internal diffusion after the sintering process of all fabricated SSLBs.

**Figure 2:**
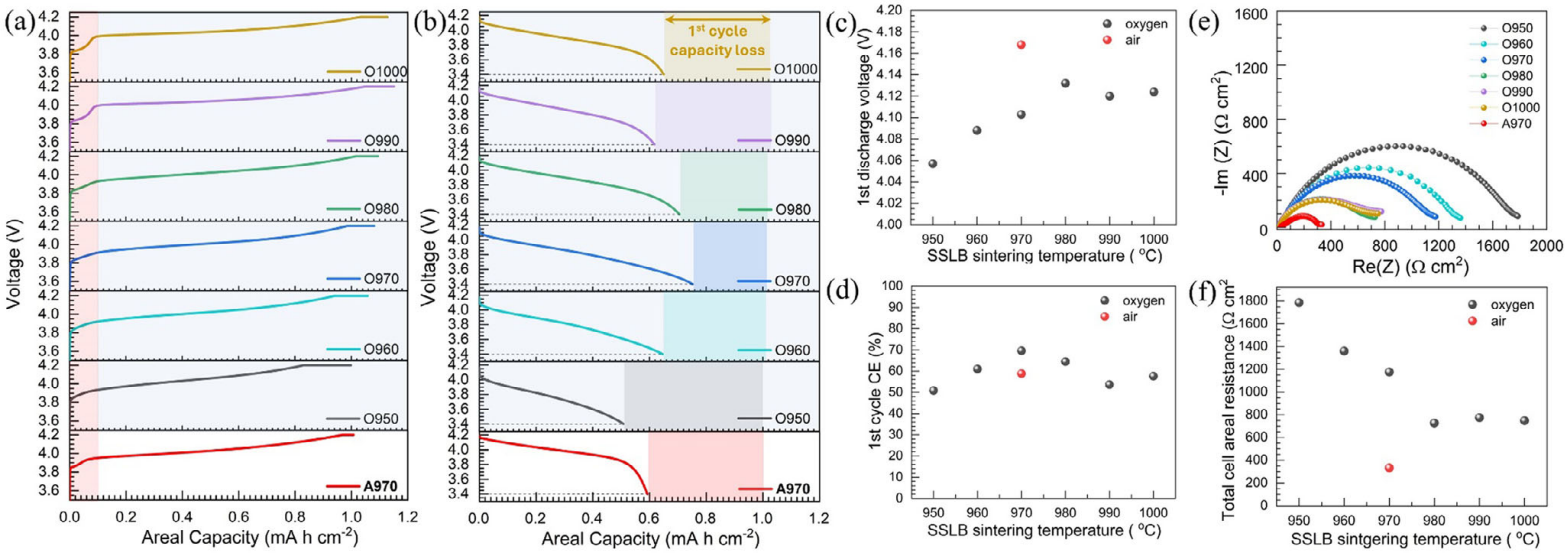
First electrochemical cycle performance of all the SSLBs. a) first charge curves b) first discharge curves, c) first discharge voltage points, d) Coulombic efficiencies for first electrochemical cycle, e) EIS results after first charge and f) total cell resistance estimated from EIS for all SSLBs.

After ensuring the phase composition, electrochemical tests were performed to understand the impact of sintering temperatures on the SSLB performance. Here, we would like to emphasize that all the SSLBs are tested without any additional liquid or polymer electrolytes and under a very low pressure of 10 N cm^−2^. **Figure** **2**a shows the first charge curves of all fabricated SSLBs. For SSLBs sintered in a pure oxygen environment, higher temperature generally leads to higher areal charge capacity, which is ≈13% difference in areal capacity between the highest (O990) and the lowest (O950). It is interesting to note that a plateau between 3.8 and 4.0 V vs. Li/Li^+^ was developed by increasing the sintering temperature from 950 to 1000 °C, which can be clearly identified at a sintering temperature higher than 980 °C. The same plateau was also observed in the first charge curve for the reference SSLB, i.e. A970. Previous research attributed this plateau to the electrochemical decomposition of impurities, such as LiOH, Li_x_O_y_, and Li_2_CO_3_, on the surface of LLZTO formed by Li‐proton exchange during the cooling process of the SSLB sintering.^[^ ^30^, ^52^ ^]^ However, Li‐proton exchange can be excluded due to the lack of H_2_O when sintering in pure O_2_ atmosphere, while further reaction of LiOH and CO_2_ to form Li_2_CO_3_ can also be excluded due to the lack of access of CO_2_. Furthermore, the plateau should also be observed in all SSLBs if it is caused by the unavoidable Li_x_O_y_ on the surface of LLZTO and LCO. Din et al. recently observed that Co incorporated Ga‐substituted LLZO shows a non‐reversible oxidation reaction at ≈4.0 V vs. Li/Li^+.[^ ^53^ ^]^ They hypothesize that the oxidation of incorporated Co^2+^ to Co^3+^ during the cyclic voltammetry oxidation process leads to the destabilization of LLZO to form a stable interphase. Considering this plateau contributes a capacity of 0.09 mAh cm^−2^ for O1000, which is ≈8% of the total charge capacity, one can approximately estimate the thickness of the Co incorporated layer in the LLZTO particles in the composite cathode to be 130 nm by simply assuming that the LLZTO particles are round shape 2 microns in diameter, roughly estimated from SEM image, and 30.7 mA h g^−1^ of specific capacity for one Li‐ion storage in LLZTO based on Co^2+^/Co^3+^ redox reaction when one Co is substituted to one Li‐sites of LLZTO per formula. The much smaller contribution of this plateau to the SSLBs sintered below 970 °C suggests that the Co diffusion can be well controlled by optimizing sintering conductions.

The first discharge curves of the fabricated SSLBs are shown in Figure 2b. Compared with A970, most of the SSLBs sintered in pure O_2_ atmosphere exhibited higher first discharge areal capacities, except for O950. Assuming that all the SSLBs reach the equilibrium charging potential at 4.2 V vs. Li/Li^+^ during the constant‐current‐constant‐voltage charging process, the first discharge voltage points can be used as an indication of the total cell resistance of each SSLB, Figure 2c. The voltages of the first discharge voltage points increase with increasing sintering temperature up to 980 °C and then decrease slightly with further increase of sintering temperature. The increase of the first discharge voltage points from 950 to 980 °C could be attributed to the better sintering of the composite cathode due to the higher sintering temperature, while the decrease of the first discharge voltage points for O990 and O1000 could be a result of the formation of Co‐incorporated interphase on LLZTO in the composite cathode, as in agreement with the observation of the extra plateau between 3.8 and 4.0 V vs. Li/Li^+^. However, the SSLBs sintered in pure oxygen atmosphere all show much lower first discharge voltage points than that for A970, suggesting that the total cell resistance of the SSLBs sintered in pure oxygen atmosphere is much higher than that of the SSLB sintered in air, i.e. A970. The CEs for the first electrochemical cycle were calculated for all the SSLBs, Figure 2d. The CE for the first electrochemical cycle improved with the increase of the sintering temperature of the SSLB under pure oxygen atmosphere, peaking at 970 °C for 69.6%. Further increase of the sintering temperature resulted in the decrease of first electrochemical cycle CE. Compared with A970, which has only 58.8% CE for its first electrochemical cycle, the CE of the first electrochemical cycle can be improved by sintering the SSLB under a pure oxygen atmosphere. Nevertheless, the dramatic capacity loss for the first electrochemical cycle still indicates a severe structural degradation during the first charging process.

Electrochemical impedance spectroscopy (EIS) was used to measure the SSLB resistances after charging to 4.2 V vs. Li/Li^+^, Figure 2e, which mainly shows a deformed semicircle for each SSLB. Here, we can assume that the interfacial resistances between Li anode and LLZTO SE in all the SSLBs were alike since the preparation process is identical, while the contribution from LLZTO solid electrolytes in all the SSLBs was all the same because the thickness of the solid electrolytes was controlled to be similar. Therefore, the differences in SSLB cell resistances should mainly come from the composite cathode that underwent different sintering conditions. The impedance of O950 reached as high as1800 Ω cm^2^ and gradually decreased to ≈750 Ω cm^2^ when the sintering temperature increased to 980 °C, Figure 2f. Further increase of the sintering temperature from 980 to 1000 °C did not substantially alter the cell resistances. It should be noted that all SSLBs sintered in pure oxygen atmosphere show much higher cell resistance than the SSLB sintered in air, which was 330 Ω cm^2^ for A970. The measured SSLB cell resistances from EIS are in agreement with the first discharge voltage points in Figure 2c,e. Nevertheless, the CE for each SSLB does not seem to correlate with the cell resistance, but was optimized at a sintering temperature close to 970 °C, Figure 2d.

**Figure 3:**
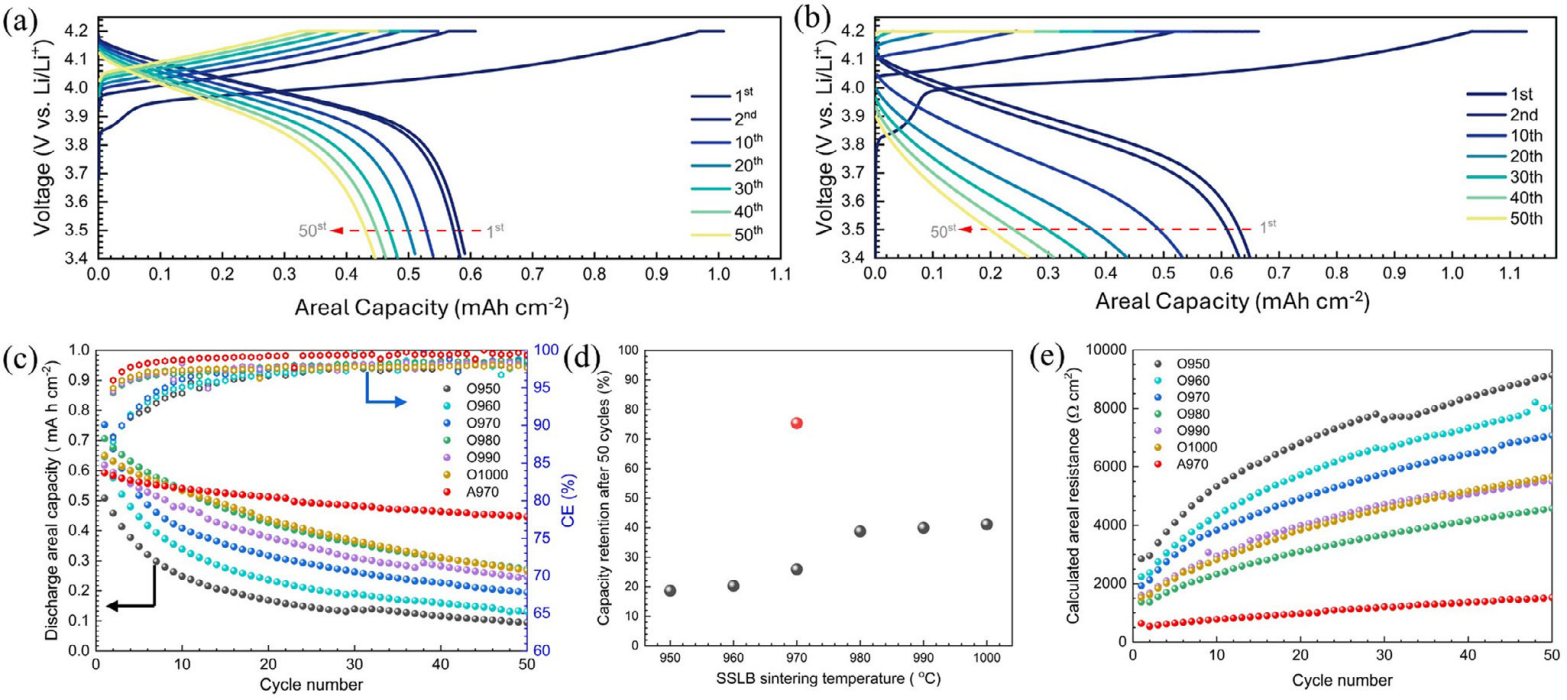
Long‐term electrochemical cycle performance of all SSLBs with a current of 50 µA cm^−2^ at 60 °C between 3.4 and 4.2 V *vs*. Li/Li^+^. Long‐term charge/discharge curves for a) A970 and b) O1000. c) long‐term cycle performance of the discharge areal capacity and Coulombic efficiency versus cycle number d) capacity retention after 50 cycles and e) calculated cell resistance versus cycle number for all SSLBs.

Long‐term electrochemical cycling was performed to investigate the stability of SSLBs sintered at different temperatures in pure oxygen atmosphere for comparison with that sintered in air at 970 °C, i.e. A970. The long‐term charge/discharge curves for A970 and O1000 are shown individually in **Figure** **3**a,b, and the other SSLB cycling results are supplemented in Figure S6 (Supporting information). Comparing the two SSLBs, A970 shows the typical LCO charge/discharge behavior in its first cycle, where the discharge curve shows a steep drop when reaching ≈3.8 V *vs*. Li/Li^+^, while that for O1000 is more rounded in shape, but also shows the steep drop when reaching ≈3.6 V *vs*. Li/Li^+^. The difference in the shape of the A970 and O1000 discharge curves can be understood from the much higher cell resistance of O1000 (749 Ω cm^2^) than that of A970 (332 Ω cm^2^) in Figure 2e,f. As the number of cycles increases, the discharge curves of O1000 change from convex to linear and then to concave, indicating the rapid development of cell resistance during cycling. The developed high cell resistance can also be seen in the charge curve of O1000 where it reaches the cut‐off voltage of 4.2 V vs. Li/Li^+^ immediately after the application of the charge current at the 40th cycle. Furthermore, it is worth noting that the plateau between 3.8 and 4.0 V vs. Li/Li^+^ is only observed in the first charge curve for both A970 and O1000. This suggests that the material can be delithiated but is no longer able to be lithiated again with a high possibility of being electrochemically decomposed. This agrees with the report from Din et al.^[^ ^53^ ^]^ It is interesting to note that a 130 nm thick delithiated interphase layer does not obviously contribute to the cell resistance as shown in Figures 2e and 3e, when compared to the lower temperature sintered SSLBs. This implies that the decomposition of Co‐substituted LLZTO into Li‐ion insulating phases at the interface as interphases during the first charge is unlikely. Furthermore, the very similar first and second discharge curves for both A970 and O1000 indicate that the cell resistance increases dramatically only during the first charge cycle, which could also be related to the microstructural cracking.

Figure 3c shows the areal capacities and CEs of the tested SSLBs vs. the number of cycles. The reference battery A970 shows the best long‐term cycling stability among all these batteries. Although the CE for its first cycle was only 58.8%, it rapidly increased to 96% for the second cycle and higher than 99% for the 15th cycle. After 50 cycles, A970 retained 75.4% of its initial capacity, Figure 3c. In contrast to A970, the capacity retentions after 50 cycles for SSLBs sintered in pure oxygen atmosphere increased with increasing sintering temperatures, but showed much lower capacity retentions than that for A970, only 41.1% for the highest performance O1000 and 18.6% for the lowest performance O950, Figure 3d. Although O970 had 11% higher CE for its first cycle than that of A970, Figure 2d, the CE for the second cycle of O970 only reached 88%, which is ≈8% lower than that of A970. The CE increased only slowly from 88% to 98% at the 30th cycle, but never reached 99% within the tested 50 cycles, Figure 3c. A similar trend of low CEs was also observed for O950 and O960 in their long‐term tests. For O980, O990, and O1000, their second cycle CEs were higher than the others, reaching 95%, which is similar to that of A970. However, their overall 50 cycle CEs also stayed below 99%, the same as that of O950, O960, and O970, which leads to the low capacity retention after 50 cycles.

Assuming that all SSLBs reach 4.2 V vs. Li/Li^+^ after charging, the total cell resistance of the SSLBs can be calculated as a function of the number of cycles using Equation (1):

(1) $$R=\frac{\Delta U}{I}$$

where *ΔU* is the voltage drop between the measured first discharge voltage data point and the equilibrium voltage 4.2 V, *I* is the applied current density, and *R* is the total resistance. The calculated resistance of A970 increased slowly from 638.4 to 1522 Ω cm^2^ after 50 cycles. Although O980 has the lowest impedance for the entire long‐term cycle among the SSLBs sintered in pure oxygen atmosphere, from 1370.6 to 4573.2 Ω cm^2^, its impedance growth is still much faster than that of A970. The much faster development of cell resistance for SSLBs sintered in pure oxygen atmosphere than that sintered in air suggests that the degradation of the composite cathode sintered in pure oxygen atmosphere is faster than that sintered in air.

**Figure 4:**
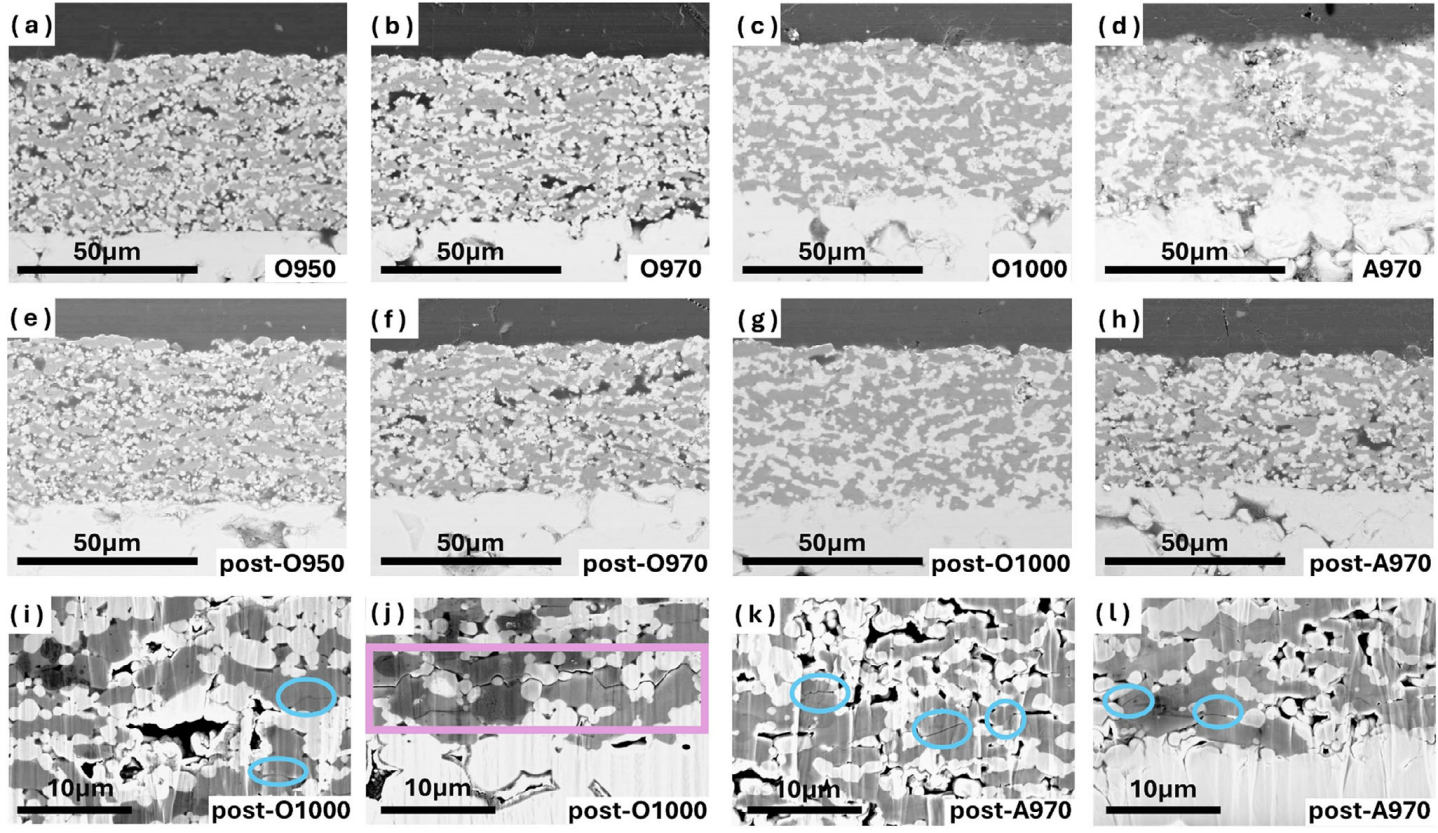
SEM images of the composite cathode microstructures from before electrochemical cycling of a) O950, b) O970, (c) O1000, and d) A970; after electrochemical cycling of e) O950, f) O970, g) O1000 and h) A970. High magnification SEM images of the composite cathode microstructure of electrochemically cycled i) outer part and j) inner part to SE of O1000 and k) outer part and l) inner part to SE of A970.

The microstructure of the composite cathodes after sintering and electrochemical cycling was investigated by SEM, **Figure** **4**. The composite cathodes for O950, O970, and O1000 were selected to show the influence of sintering temperatures on the microstructures, as shown in Figure 4a–c. A higher sintering temperature facilitates the sintering process to obtain a denser microstructure of the composite cathode, as supported by the porosity calculation in Figure S7 (Supporting information), and also a better contact between the composite cathode and the SE. In the composite cathode of O950, Figure 4a, many pores can be seen within the composite cathode, ranging in size from submicrometer to tens of micrometers, and many voids can be seen at the interface between the composite cathode and the SE. These pores and voids are obstacles for the ionic and electronic conduction. The detour of ionic and electronic conduction paths, i.e. higher tortuosity for conduction paths, causes large impedance of the cell. The overall structure becomes denser when the sintering temperature increases to 970 °C, Figure 4b, while some pores are still observed. The denser microstructure reduces the length of diffusion paths for Li‐ions and electrons to reduce the cell resistance. When the sintering temperature was increased to 1000 °C, Figure 4c, the composite cathode shows the densest microstructure. The contact necks between LCO and LLZTO are well established, and only a few sub‐micro pores remain between the LCO and LLZTO, which are almost invisible at this magnification. The composite cathode and SE are also tightly bonded, with only a few voids left at where the defects of the SE surface are located. The improvement of the microstructure with increasing sintering temperature explains the decreasing impedance of the cell, as illustrated by Figure 2e,f. As shown in Figure 4d, the reference battery A970 has a microstructure similar to that of the O970. However, the A970 has a much lower cell impedance, 331 vs. 1176 Ω cm^2^, and better electrochemical performance, 75.4 vs. 25.9% capacity retention after 50 cycles, than that of O970. The results directly indicate the effect of the sintering atmosphere on the electrochemical performance of SSLBs.

The microstructure of the composite cathodes after cycling was also investigated by SEM, Figure 4e–h. Although the high porosity of the sintered composite cathode for O950 makes it difficult to find evidence of structural degradation after electrochemical cycling, LLZTO particles were pulverized into smaller ones, and a few trans‐granular cracks across LCO particles were identified due to repeated volume expansion and contraction of LCO during electrochemical cycling, Figure 4e and Figure S8 (Supporting information). Similar structural degradation was also observed for cycled O970 and A970, Figure 4f,h. Moreover, the structural degradation is more apparent as more pores appear in the post‐cycled composite cathodes for O970 and A970 when compared to the before cycled ones. The increase in porosity could be a result of the detachment of pulverized LLZTO and/or LCO particles during the polishing. This is because the lower porosity of the composite cathodes for O970 and A970 did not allow the epoxy to infiltrate and retain these pulverized particles. Such loosely packed pulverized particles mean that the diffusion pathways for electrons and ions have been disrupted during cycling, which leads to an increase in cell impedance and loss of capacity. This is regarded as one of the main reasons for the capacity degradation of the garnet structure‐based SSL.^[^ ^29^ ^]^ For the O1000 composite cathode, the overall microstructure after cycling was still very dense, as only a small number of pores appeared, and most of them are only submicron in size, Figure 4g,i. However, higher magnification SEM images reveal not only trans‐granular cracks within some LCO particles but also inter‐granular cracks that are tens of micrometers long near the bottom part of the composite cathode of O1000, Figure 4i,j. Such kind of long inter‐granular cracks should appear due to a lack of free space in this highly dense and rigid microstructure to relieve the stress induced by the volume change of LCO during cycling. The long inter‐granular cracks explain the low CE and low‐capacity retention of O1000 due to the dramatic increase in ionic and electronic diffusion paths, i.e. higher resistance or loss of contact with the LCO. In contrast to SSLBs sintered in pure oxygen atmosphere, the composite cathode microstructure of A970 shows only some inter‐granular cracking of LCO and the increase of porosity due to pulverization of LCO/LLZTO particles, Figure 4k,l, which explains the higher long‐term electrochemical cycling stability of A970.

To study the impact of oxygen vacancy in LCO on the performance of garnet‐based SSLBs, the LCO/LLZTO composite cathode was sintered in a pure oxygen atmosphere to limit the formation of oxygen vacancy as much as possible and compared with that sintered in air. In addition, a temperature‐dependent sintering study was carried out to optimize the SSLB performance to limit the influence of using a pure oxygen atmosphere for sintering. From the microstructure of their composite cathodes, the sintering atmosphere seems to have no discernible impact, but their electrochemical performances are very different. Especially, the cell resistances for those SSLBs sintered in pure oxygen atmosphere are all much higher than that sintered in air. As LCO is known to be a p‐type semiconductor,^[^ ^54^ ^]^ the formation of oxygen vacancies during high temperature sintering increases the concentration of electronic holes, which subsequently increases the conductivity of LCO. The sintering of LCO/LLZTO composite cathode in a pure oxygen atmosphere has, therefore, an effect to reduce the LCO conductivity. Also, LCO sintered in a pure oxygen atmosphere restores LCO to its stoichiometric form by reducing defects and oxidizing cobalt to predominantly Co^3+^. The reduction of mixed valence states, i.e. the Co^2+^/Co^3+^ ratio, where Co^2+^ originates from oxygen vacancy formation to keep the system neutral, where electrons can hop between Co^2+^ and Co^3+^ sites, further reduces the electronic conductivity.^[^ ^55^ ^]^ This explains the measured higher impedance for SSLB sintered in a pure oxygen atmosphere. Besides the higher cell resistance for SSLBs sintered in pure oxygen atmosphere, the long‐term cycling performances for them are also not as good as that sintered in air. The higher capacity degradation for SSLBs sintered in the pure oxygen atmosphere implies either that the volume change of LCO is larger or the segregation of Co from LCO is more severe during electrochemical cycling with less oxygen vacancies in LCO. The results suggest that sintering LCO/LLZTO composite cathode in pure oxygen atmosphere only brings drawbacks without apparent advantage due to the dramatically higher resistance of LCO, which should be avoided for this type of SSLB fabrication. Although a higher relative density of the composite cathode means higher volumetric energy density of the SSLB, we observed that a high relative density microstructure for the composite cathode, e.g. O1000, is not always beneficial for garnet‐type SSLB due to the lack of free space for stress relief during electrochemical cycling, which subsequently leads to large inter‐granular cracks to lower the capacity retention. Careful microstructural engineering is necessary to further improve the long‐term cycle stability of this type of SSLB.

Furthermore, the SSLBs sintered in pure oxygen atmosphere also formed an additional plateau between 3.8 and 4.0 V vs. Li/Li^+^ during the first charge cycle when the sintering temperature is higher than 970 °C and becomes very pronounced at 990 and 1000 °C. Previous studies have attributed this plateau to the electrochemical decomposition of Li_2_CO_3_ and/or LiOH.^[^ ^52^, ^56^ ^]^ However, the high purity oxygen (99.9992%) from compressed gas bottle did not provide sufficiently high levels of water and CO_2_ for Li‐proton exchange to result in the formation of Li_2_CO_3_ and LiOH that would contribute a capacity of 0.09 mAh cm^−2^ for O1000. Also, if Li_2_CO_3_, Li_2_O and LiOH would be intrinsically present in the as‐prepared LLZTO, the plateau should also be observed for SSLBs sintered at temperatures lower than 970 °C, which is not the case in this study. Therefore, the formation of this plateau agrees with the finding from Din et al. that comes from the Co interdiffusion from LCO into LLZTO, which undergoes the oxidation reaction from Co^2+^ to Co^3+^ to release one Li^+^ from LLZTO during charging. Interestingly, the non‐reversible extraction of Li^+^ from this plateau suggests that the amount of material decomposed during the first charge cycle can be estimated to be ≈130 nm in thickness of this Co‐incorporated LLZTO layer for O1000. The decomposed materials at the interface as interphases should dramatically increase the cell resistance due to blocking of ionic and/or electronic conductive pathways, but seems not to be the case in this study, e.g. the cell resistance for O1000 is not higher than that for O980 or O990 after the first charge cycle, Figure 2e. Furthermore, this Co‐incorporated LLZTO layer can be minimized by properly controlling the sintering process and temperature, as shown for the A970 or lower sintering temperature. Therefore, the optimization of the microstructure to accommodate the generated stress during electrochemical cycling seems, at least from our point of view, the most important aspect for further improvement of the electrochemical cycle stability of LLZO‐based SSLBs.

## 3 Conclusion

A series of garnet‐based SSLBs were fabricated by sintering the composite cathode in a pure oxygen atmosphere with different sintering temperatures to minimize the impact of changing the sintering atmosphere from air. Raman mapping showed that the LCO and LLZTO phases were clearly retained after the high temperature sintering process, except that O1000 contains a small amount of secondary phase, which may be Li_0.5_La_2_Co_0.5_O_4_. Together with the SEM‐EDX results, it can be concluded that there are neither severe chemical reactions nor internal elemental diffusion occur during the sintering process under the oxygen atmosphere.

Electrochemical tests show that an additional non‐reversible charging plateau between 3.8 and 4.0 V *vs*. Li/Li^+^ is observed in all SSLBs sintered at temperatures higher than 980 °C. The result excludes the previous explanation of impurity phases from proton exchange reactions, such as LiOH, Li_x_O_y_, and Li_2_CO_3_, but agrees with the finding of Din et al. that this could be caused by Co^2+^/Co^3+^ oxidation in LLZTO due to Co incorporation into LLZTO during the high temperature sintering process. Compared to A970, the SSLBs sintered in an oxygen atmosphere show much higher initial cell resistances and faster capacity degradations. The high cell resistance can be explained by the reduction of electronic holes and Co^2+^/Co^3+^ mixed valences in the LCO structure by sintering it in a pure oxygen atmosphere, which reduces the electronic conductivity of LCO. From their long‐term electrochemical cycling, the similarity of the microstructures for O970 and A970 rules out that the higher capacity degradation of O970 is from the effect of significant microstructural inconformity. Thus, the inferior electrochemical performance of the SSLBs sintered in a pure oxygen atmosphere could only be due to worse LCO properties, such as possibly to be larger volume changes upon lithiation/delithiation.

Furthermore, SEM images of cycled SSLBs show pulverization of LLZTO and LCO particles, and trans‐granular cracks of LCO particles are the reasons for the capacity degradation of all the SSLBs. For O1000, its highly dense microstructure resulting from the high sintering temperature (1000 °C) leads to long intergranular cracks during electrochemical cycling. Such severe structural damage may be due to the over‐dense microstructure being unable to relieve the stress accumulated during the electrochemical cycling. Thus, careful microstructure design is required for advancing the performance of LLZO‐based SSLBs.

## 4 Experimental Section

### Preparation of SSLB

The preparation of the SSLBs were the same as descripted in the previous work.^[^ ^29^, ^30^ ^]^ Here, only a brief description is provided.

Garnet‐type solid electrolyte Li_6.45_Al_0.05_La_3_Zr_1.6_Ta_0.4_O_12_ (LLZTO) was prepared by solid‐state reaction by using LiOH∙H_2_O (Merck, 98%), La_2_O_3_ (Merck, 99.9%, pre‐dried at 900 °C for 10 h), ZrO_2_ (Treibacher, 99.5%), Ta_2_O_5_ (Inframat, 99.95%), and 𝛼‐Al_2_O_3_ (Inframat, 99.9%). Dry mixing and crashing were carried out between the steps including calcination once at 850 °C and twice at 1000 °C for 20 h, and before sintering at 1175 °C for 10 h. After sintering, LLZTO pellets were sliced and polished to get discs with a thickness of ≈600 µm.

Composite cathode ink was prepared by ball milling with an equal mass of LCO (MTI Corp., USA) and LLZTO powders, i.e. 50:50 wt.%. Then, three‐roll milling was used for mixing 3 wt.% ethyl cellulose (Sigma–Aldrich) in terpineol (Sigma–Aldrich) and the solid loading with a weight ratio 1:1. After that, the ink was hand‐brushed onto one side of the LLZTO discs to sinter in a tube furnace (Nabertherm, Germany) under a pure oxygen and air atmosphere at required temperatures. The oxygen flow was ≈7 liters per hour. Consider pure oxygen environment may change the optimized sintering temperature of the composite cathode, 6 different temperatures, i.e. 950, 960, 970, 980, 990, and 1000 °C, were investigated while the reference SSLB was sintered in air at 970 °C. All the fabricated SSLBs have a similar composite cathode loading of ≈13 mg, which gives an LCO loading of ≈6.5 mg cm^−2^. For the anode, Au interlayer was sputtered onto LLZTO to help the Li adhesion. The fabricated SSLBs were also heated to 250 °C to further reducing the LLZTO/Li interfacial resistance before being put into a Swagelok cell for electrochemical measurements. A spring was used in the Swagelok cell to provide a pressure of 0.1 MPa.

### Electrochemical Measurements

The SSLBs were electrochemically cycled at 60 °C using a multi‐potentiostat (BioLogic VMP‐300) combined with a climate chamber (Maccor, Inc., USA). SSLBs were first charged to 4.2 V vs. Li/Li^+^ with a constant current density of 50 µA cm^−2^ and held at 4.2 V until the current dropped to 10 µA cm^−2^. Then, SSLBs were discharged to 3.4 V *vs*. Li/Li^+^ with a constant current density of 50 µA cm^−2^. Electrochemical impedance spectroscopy (EIS) measurements were conducted after the first charging of each SSLB, with frequency varied from 7 MHz to 1 Hz and a perturbation AC amplitude of 10 mV.

### Sample Characterizations

The Raman spectroscopy mappings were carried out with a WITec (Ulm, Germany) Alpha 300R Raman microscope using a 532 nm excitation laser with 1.0 mW of power and 600 g mm^−1^ grating. Before the mappings, the samples were immersed in resin and then polished with SiC sandpaper to get a flat cross‐section. The spectra were collected for an area of 25 µm × 20 µm by using a Zeiss EC Epiplan‐Neofluar 100×/0.9 DIC objective. A step size of (x, y) = (0.357, 0.5 µm) and a 3‐s spectral acquisition time were chosen for maximal resolution and minimal time per spectrum. The total number of acquired spectra for the mapping was 2800. The mapping was analyzed utilizing the TrueComponent Analysis within the WITec Project 6.2 software, which finds components and creates intensity distribution images of the components.

To investigate the microstructure and elemental distributions, SEM and EDX experiments were conducted using a Quanta FEG 650 (FEI) environmental scanning electron microscope matched with EDAX APEX. All the investigations were performed with the e‐beam at a voltage of 20 kV.

## Conflict of Interest

The authors declare no conflict of interest

## Author Contributions

All authors made contributions to manuscript preparation and have given final approval for publication.

## Supporting information



Supporting Information

## Data Availability

The data that support the findings of this study are available from the corresponding author upon reasonable request.
