# Supplementary material for: Impact of Oxygen Vacancies in LiCoO2 on the Electrochemical Performance of Garnet‐Based All‐Solid‐State Li‐Metal Batteries
Source: Adv Sci (Weinh). 2025 Jul 25;12(39):e08750. doi: 10.1002/advs.202508750 (PMC12533151; doi:10.1002/advs.202508750)
Supplement: Supplementary file 1 — Supporting Information [file ADVS-12-e08750-s001.docx]

Supporting Information

Impact of Oxygen Vacancy in LiCoO_2_ on the Electrochemical Performance of Garnet-Based All-Solid-State Li Metal Batteries

Zhizhen Qin ^a,b^, Jehad Ahmed ^a,b^, Sebastian Speer ^a,b^, Dmitri L. Danilov ^a,c,^*, Sven Jovanovic ^a^, Anna Windmüller ^a^, Shicheng Yu ^a^, Chih-Long Tsai ^a,^*, Hermann Tempel ^a^, Wen-Wei Wu^d^, Jeng-Kuei Chang^d^, Rüdiger-A. Eichel ^a,b,e^ and Peter H.L. Notten ^a,c,d^

^a^ Institute of Energy Technologies: Fundamental Electrochemistry (IET-1), Forschungszentrum Jülich GmbH, 52428, Jülich, Germany.

^b^ Institute of Physical Chemistry, RWTH Aachen University, D-52074 Aachen, Germany.

^c^ Eindhoven University of Technology, P.O. Box 513, 5600 MB Eindhoven, The Netherlands.

^d^ Department of Materials Science and Engineering, National Yang Ming Chiao Tung University
Hsinchu 30010, Taiwan

^e^ Institute of Energy Materials and Devices, Helmholtz Institute Münster: Ionics in Energy Storage (IMD-4/HI MS), Forschungszentrum Jülich, 48149 Münster, Germany

^f^ University of Technology Sydney, Broadway, Sydney, NSW 2007, Australia

^*^ Correspondence:

Chih-Long Tsai

Email: c.tsai@fz-juelich.de

Dmitri L. Danilov

Email: d.danilov@fz-juelich.de


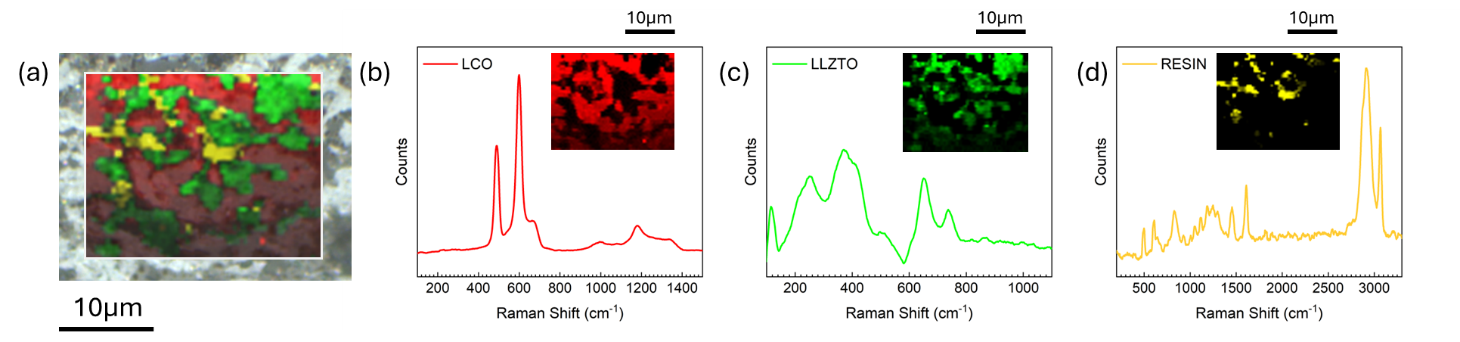


**Figure S1.** Raman mapping of the sintered composite cathode for A970, (a) optical image overlay with Raman mapped area and (b)-(d) analyzed average Raman spectra for LCO, LLZTO, and resin, respectively.


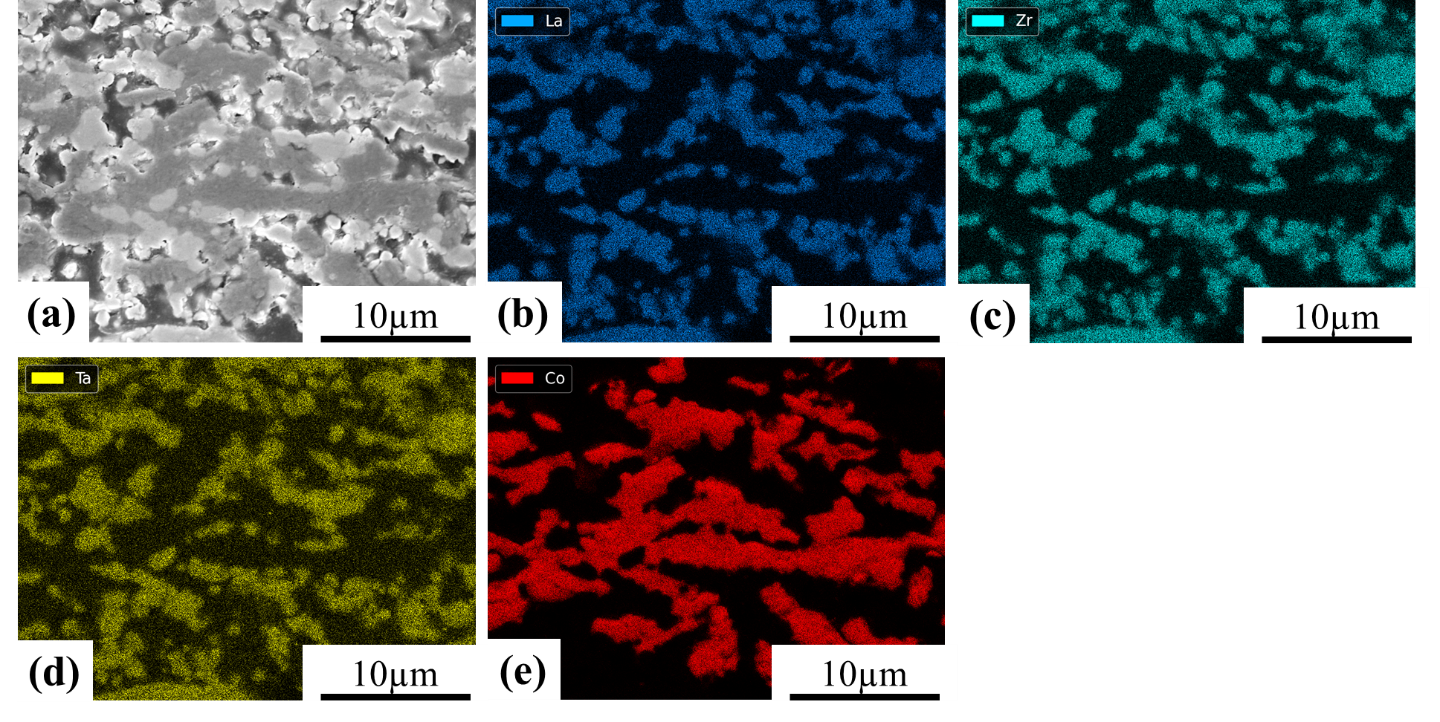


**Figure S2.** SEM-EDS mapping of O950 after sintering, (a) SEM image; element distribution of (b) La, (c) Zr, (d) Ta, and (e) Co.


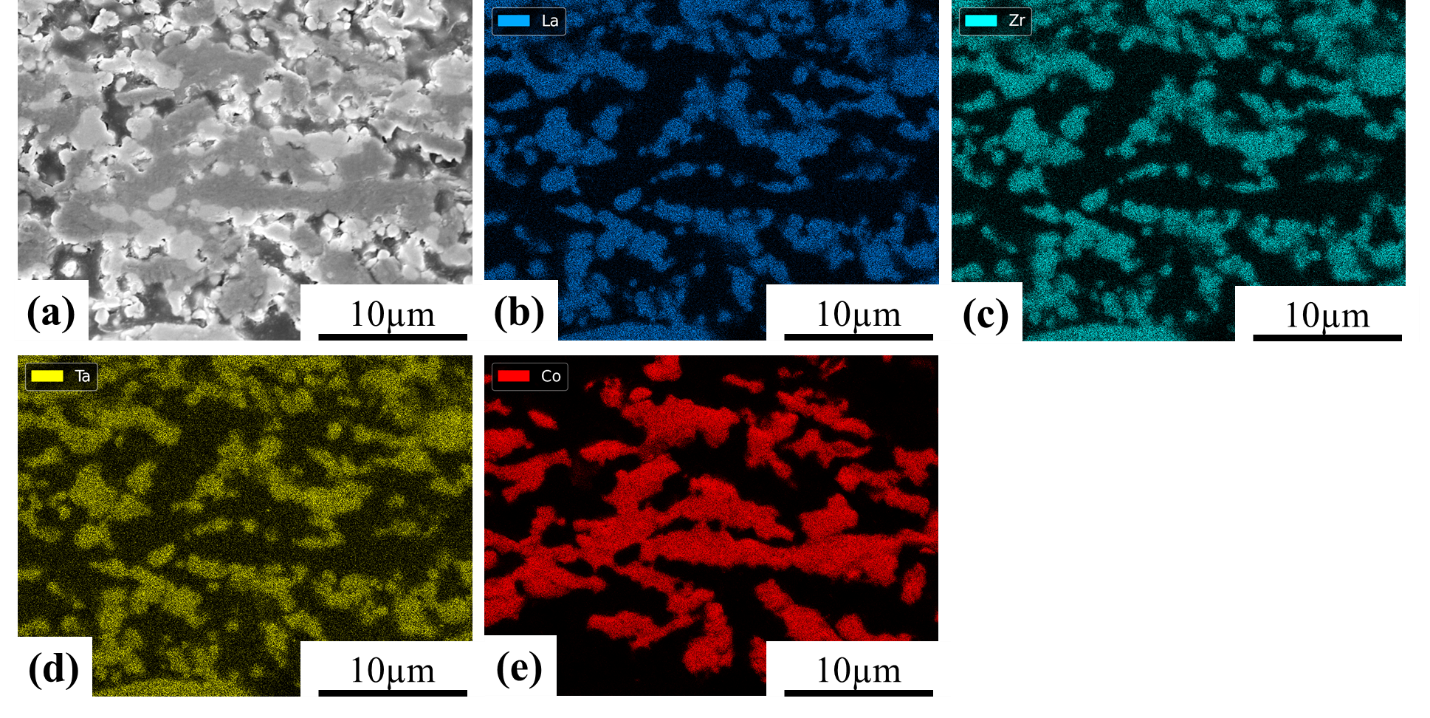


**Figure S3.** SEM-EDS mapping of O970 after sintering, (a) SEM image; element distribution of (b) La, (c) Zr, (d) Ta, and (e) Co.


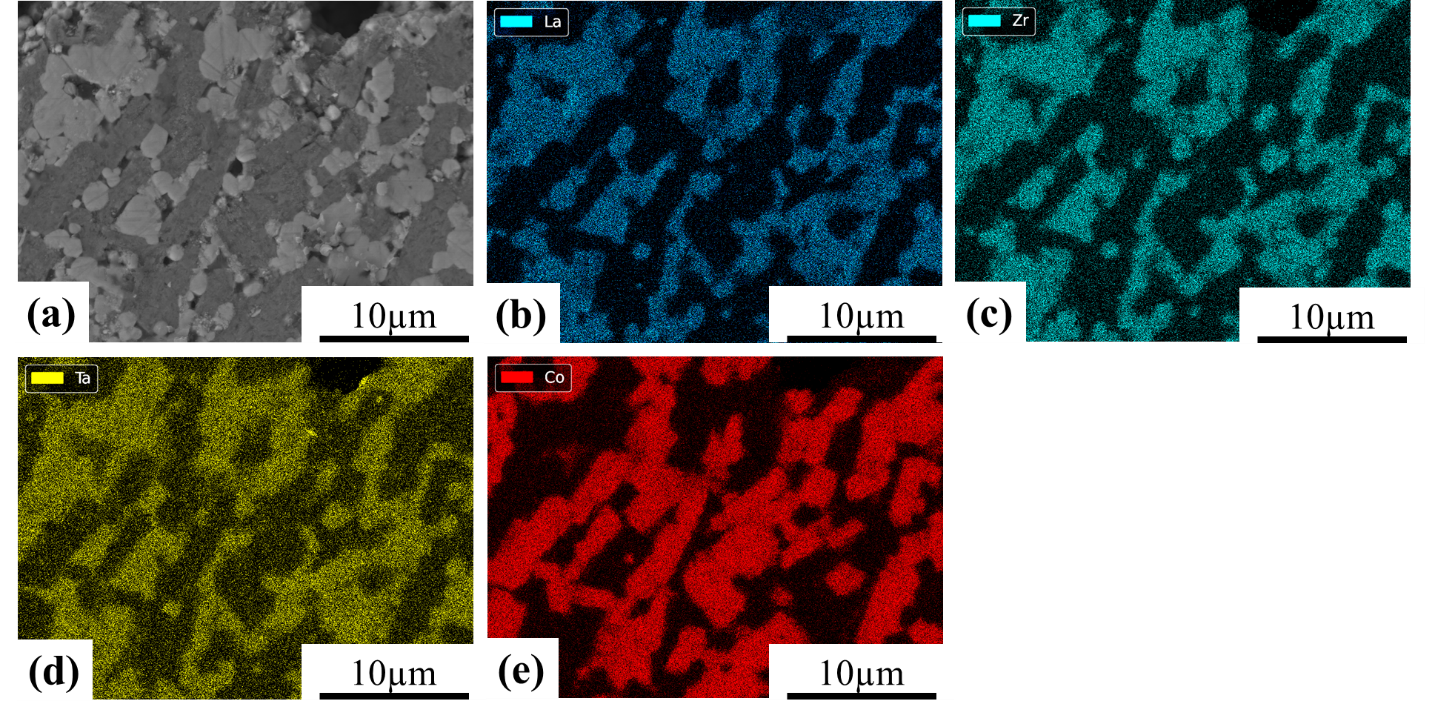


**Figure S4.** SEM-EDS mapping of O1000 after sintering, (a) SEM image; element distribution of (b) La, (c) Zr, (d) Ta, and (e) Co.


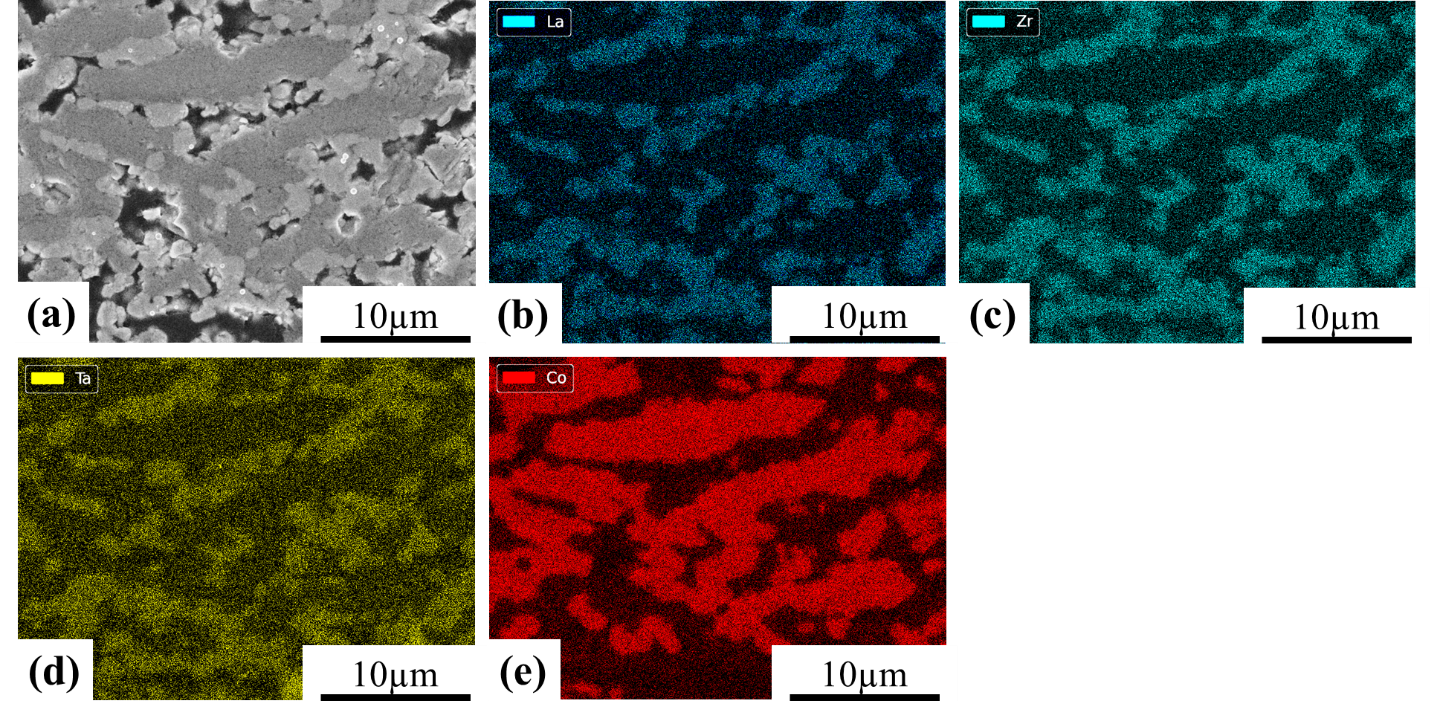


**Figure S5.** SEM-EDS mapping of A970 after sintering, (a) SEM image; element distribution of (b) La, (c) Zr, (d) Ta, and (e) Co.


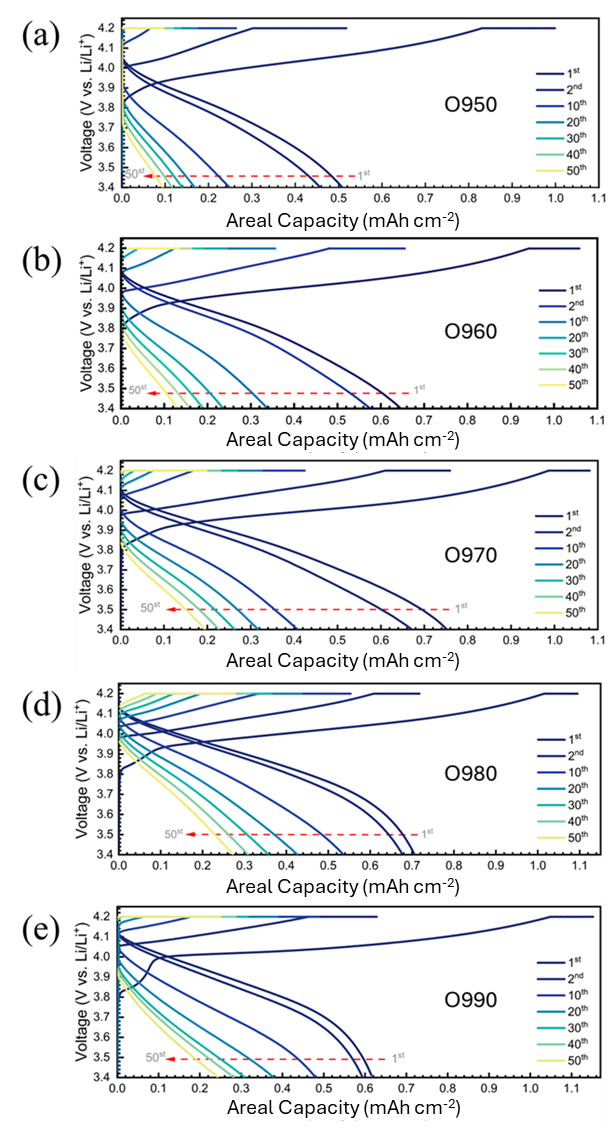


**Figure S6.** Long-term electrochemical cycle performance of SSLBs with a current of 50 μA cm ^-2^ at 60 ^o^C between 3.4 and 4.2 V vs. Li/Li^+^. Long-term charge/discharge curves for (a) O950, (b) O960, (c) O970 (d) O980, and (e) O990.


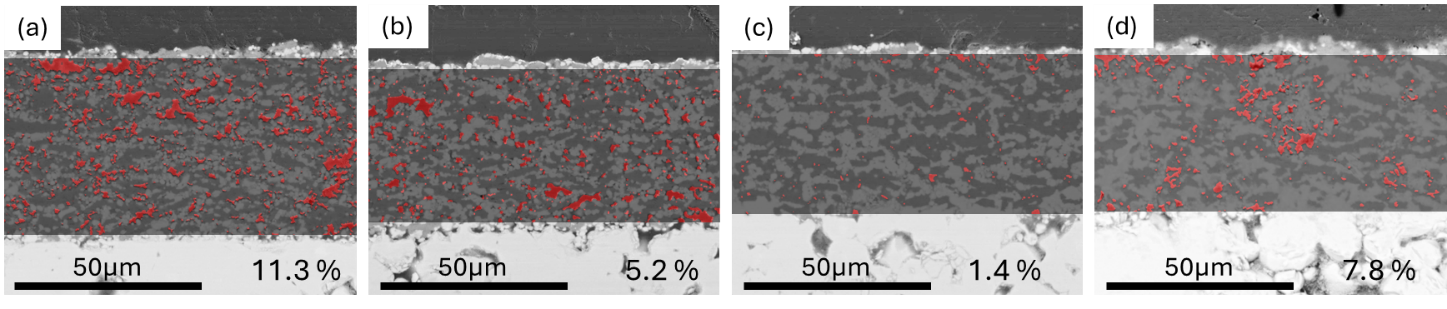


**Figure S7.** Porosity of different CPEs (a) O950, (b) O970, (c) O1000 and (d) A970 before cycling.

The porosity of composite cathode was obtained by processing the SEM image by Python. First, a Gaussian filter was applied to SEM image to reduce noise, then a Kuwahara filter was applied to enhance the edges between phases. Subsequently, threshold-based segmentation was performed to segment the pores and calculate the area percentage. The porosity of the composite cathode can reflect its density. A higher porosity represents a lower density. As shown in Figure S7(a-c), the porosity of composite cathode sintered in oxygen atmosphere decreases significantly with increasing sintering temperature thus we can make a conclusion that the density of the composite cathode increases with increasing sintering temperature. Besides, the density of O1000, Figure S7(c), is also clearly larger than A970, Figure S7(d), which means O_2_ sintering atmosphere will not bring significant drawbacks on the microstructure.


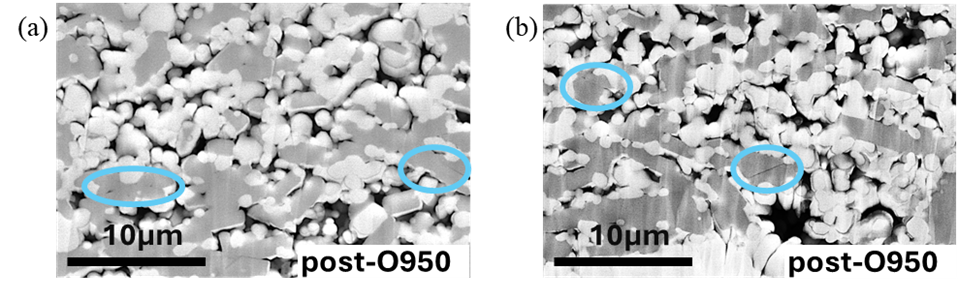


**Figure S8.** High magnification SEM images of the composite cathode microstructure of electrochemically cycled (a) outer part and (b) inner part of O950. Blue ovals point to the trans-granular cracks.
